# Effects of Obesity on Bone Mass and Quality in Ovariectomized Female Zucker Rats

**DOI:** 10.1155/2014/690123

**Published:** 2014-09-18

**Authors:** Rafaela G. Feresin, Sarah A. Johnson, Marcus L. Elam, Edward Jo, Bahram H. Arjmandi, Reza Hakkak

**Affiliations:** ^1^Department of Nutrition, Food and Exercise Sciences, Florida State University, Tallahassee, FL 32306, USA; ^2^The Center for Advancing Exercise and Nutrition Research on Aging (CAENRA), Florida State University, Tallahassee, FL 32306, USA; ^3^Department of Kinesiology and Health Promotion, California State Polytechnic University, Pomona, CA 91768, USA; ^4^Department of Dietetics and Nutrition, University of Arkansas for Medical Sciences, Little Rock, AR 72202, USA; ^5^Department of Pediatrics, University of Arkansas for Medical Sciences, Little Rock, AR 72202, USA; ^6^Arkansas Children's Hospital Research Institute, Little Rock, AR 72202, USA; ^7^Arkansas Cancer Research Center, Little Rock, AR 72205, USA

## Abstract

Obesity and osteoporosis are two chronic conditions that have been increasing in prevalence. Despite prior data supporting the positive relationship between body weight and bone mineral density (BMD), recent findings show excess body weight to be detrimental to bone mass, strength, and quality. To evaluate whether obesity would further exacerbate the effects of ovariectomy on bone, we examined the tibiae and fourth lumbar (L4) vertebrae from leptin receptor-deficient female (*Lepr* ^*fa*/*fa*^) Zucker rats and their heterozygous lean controls (*Lepr* ^*fa*/+^) that were either sham-operated or ovariectomized (Ovx). BMD of L4 vertebra was measured using dual-energy X-ray absorptiometry, and microcomputed tomography was used to assess the microstructural properties of the tibiae. Ovariectomy significantly (*P* < 0.001) decreased the BMD of L4 vertebrae in lean and obese Zucker rats. Lower trabecular number and greater trabecular separation (*P* < 0.001) were also observed in the tibiae of lean- and obese-Ovx rats when compared to sham rats. However, only the obese-Ovx rats had lower trabecular thickness (Tb.Th) (*P* < 0.005) than the other groups. These findings demonstrated that ovarian hormone deficiency adversely affected bone mass and quality in lean and obese rats while obesity only affected Tb.Th in Ovx-female Zucker rats.

## 1. Introduction

Despite considerable public health efforts to curtail obesity, the epidemic has progressed over the past three decades in the United States (US). Epidemiological data from the most recent National Health and Examination Survey (NHANES 2009-2010) indicated that 68.8% of American adults are overweight or obese (body mass index, BMI ≥ 25 kg/m^2^). Nearly half of this population has a BMI ≥ 30 kg/m^2^, suggesting that about 36% or one-third of the US adult population is currently obese. While obesity has been linked to a number of chronic diseases, overwhelming epidemiological data indicate a positive correlation between BMI and BMD [1–6].

Although the exact mechanisms underlying this observation are not fully understood, this protective effect could be attributed to increased mechanical load, imposed by a greater weight on weight-bearing bones as well as hormonal changes associated with obesity, for example, increased synthesis of estrogen and leptin by adipocytes. Higher levels of estrogen, known to suppress osteoclastic bone resorption and stimulate osteoblastic bone formation, have been found in serum of obese postmenopausal women when compared to their normal weight counterparts [7]. This fact is attributed to an increased peripheral production of estrone through increased aromatization of androstenedione by aromatase in the white adipose tissue [8, 9]. Additionally, plasma leptin levels have been directly associated with the amount of fat mass [10]. Though leptin is primarily known to influence energy intake and expenditure, it has also been shown to be an important factor in the regulation of bone remodeling. Thomas et al. [11] reported that leptin promotes osteoblastogenesis and inhibits adipogenesis in human marrow stromal cells. In contrast to the putative protective effects of excess body weight on BMD, evidence challenging this relationship has recently emerged. Zhao et al. [12] showed that in healthy adults greater fat mass negatively correlates with BMD after correction for the mechanical loading effect of body weight. In addition, Blum et al. [13] found that serum leptin is negatively associated with BMD in premenopausal women. Further, Ducy et al. [14] observed that intracerebroventricular infusion of leptin reduced bone mass in* ob/ob* and wild-type mice, respectively.

Due to the increasing incidence and prevalence of obesity and the controversial findings in the literature about the relationship between BMI and BMD, the main purpose of this study was to investigate the effects of obesity on bone mass and quality in female Zucker rats, the most commonly used rat model of genetic obesity. Because postmenopausal women experience rapid bone loss and are prone to gaining excess body weight, we also investigated the effect of obesity on BMD in a rat model of postmenopausal bone loss.

## 2. Methods

### 2.1. Animals and Diets

The animal protocol for this study was approved by the Institutional Animal Care and Use Committee of the University of Arkansas for Medical Sciences. Six-week old leptin receptor-deficient female (*Lepr* ^*fa*/*fa*^) Zucker rats and their heterozygous lean controls (*Lepr* ^*fa*/+^) were sham-operated (lean-*Lepr* ^*fa*/+^, *n* = 6; obese-*Lepr* ^*fa*/*fa*^, *n* = 6) or Ovx (lean-*Lepr* ^*fa*/+^, *n* = 6; obese-*Lepr* ^*fa*/*fa*^, *n* = 6) by Harlan Industries (Indianapolis, IN, USA) and housed in the animal facilities at the Arkansas Children's Hospital Research Institute. Rats were housed in polycarbonate cages (2/cage) and had free access to water and a semipurified AIN-93G diet (Harlan-Teklad, Madison, WI, USA) for 21 weeks.

### 2.2. Bone Densitometry

At 27 weeks, rats were sacrificed, and L4 vertebrae and tibiae were removed and cleaned of adhering tissue. L4 vertebrae were then scanned to determine bone mineral area (BMA), bone mineral content (BMC), and BMD using dual energy X-ray absorptiometry (DXA; QDR-4500A Elite; Hologic, Waltham, MA, USA) while tibiae were frozen at −20°C for microstructural analysis.

### 2.3. Microcomputed Tomography Measurements

The microarchitectural trabecular bone structure of tibiae was evaluated using microcomputed tomography (*μ*CT40, Scanco Medical, Switzerland). The tibia was scanned from the proximal growth plate in the distal direction (16 *μ*m/slice). This region included 350 images obtained from each tibia using 1024 × 1024 matrix resulting in an isotropic voxel resolution of 22 *μ*m^3^. An integration time of 70 ms per projection was used, with a rotational step of 0.36° resulting in a total acquisition time of 150 min/sample. The volume of interest (VOI) was selected as a region 25 slices away from the growth plate at the proximal end of the tibia to 125 slices. The three-dimensional (3D) images were also obtained for visualization and display. The trabecular bone morphometric parameters assessed with *μ*CT included the bone volume expressed as a percentage of total volume (BV/TV), trabecular number (Tb.N), thickness (Tb.Th), and separation (Tb.Sp). Nonmetric parameters included structure model index (SMI) which is an indicator of plate-rod arrangement of the bone structure and connectivedensity (Conn.D).

### 2.4. Statistical Analysis

A 2 × 2 (group by time) repeated measures analysis of variance (ANOVA) was used to determine differences in body weight between groups and over time while one-way ANOVA was used to determine group differences in bone parameters after treatment. In the event of a significant main effect or interaction, Tukey-Kramer post hoc test was performed for pairwise comparisons. Significant differences were determined at *P* ≤ 0.05. Values are presented as mean ± standard error of mean (SEM). Data analyses were generated using SSPS version 20.0 for Windows (SPSS Inc., Chicago, IL).

## 3. Results

### 3.1. Body Weight

All rats gained weight during the course of the experiment. Initial and final body weights are shown in Table 1. As expected, despite the similar initial body weights, Ovx rats gained significantly more weight than sham rats. Additionally, mean final body weight for obese*-*Ovx Zucker rats was significantly (*P* < 0.001) higher than in lean-Ovx Zucker rats (340 and 562 g, resp.).

### 3.2. Bone Mineral Density and Content

Notably, the mean L4 vertebral BMD was approximately 11.5% lower in Ovx rats than in the sham rats, irrespective of body weight (Table 2), whereas the mean BMC of L4 vertebrae was only significantly higher in the obese*-*sham Zucker rats compared to lean-Ovx Zucker rats (Table 2).

### 3.3. Trabecular Microstructural Properties 

**Figure 1:**
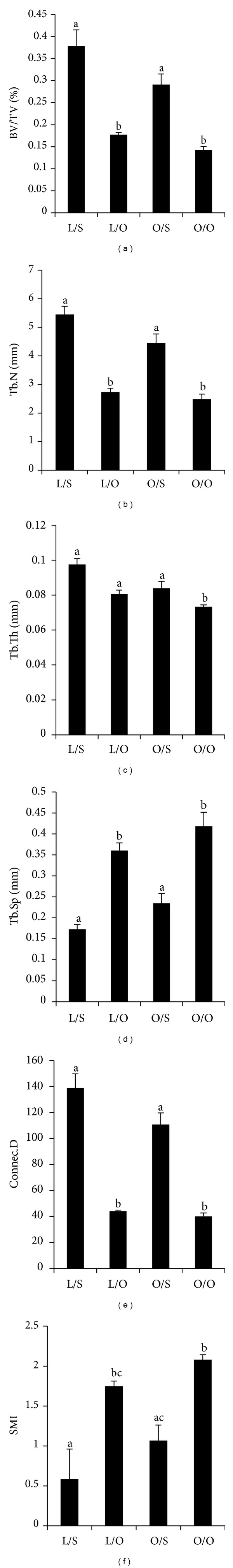
Effects of obesity and ovariectomy on tibial *μ*CT parameters in Zucker rats. Values are expressed as mean ± SEM. Values that do not share the same superscript letters are significantly (*P* < 0.05) different from each other. *n* = 6 rats/group. L/S, lean (*Lepr* ^*fa*/+^) sham-operated; L/O, lean (*Lepr* ^*fa*/+^) ovariectomized; O/S, obese (*Lepr* ^*fa*/*fa*^) sham-operated; and O/O, obese (*Lepr* ^*fa*/*fa*^) ovariectomized. (a) BV/TV bone volume as percentage of tissue volume, (b) Tb.N trabecular number, (c) Tb.Th trabecular thickness, (d) Tb.Sp trabecular separation, (e) Conn.D connective density, and (f) SMI structure model index.

The 3D image analyses of the proximal tibia indicated that ovariectomy unfavorably altered trabecular microstructural parameters in both lean and obese groups (Figure 1) with the exception of Tb.Th (Figure 1(c)). When compared to lean-sham Zucker rats, Tb.Th tended to be lower in the lean-Ovx Zucker rats (*P* = 0.06) and in the obese-sham Zucker rats (*P* = 0.09); however, this parameter was only significantly (*P* = 0.005) lower in the obese-Ovx group.

## 4. Discussion

Although obesity and osteoporosis are two chronic conditions that have long been considered to be mutually exclusive, there is evidence that a complex relationship between the two exists. For decades, an array of epidemiological studies available have reported positive correlations between BMI and/or fat mass and BMD which led to the belief that excess body weight stimulates greater bone mass, strength, and quality primarily due to mechanical loading [12, 15–17]. At the same time, several animal studies [18–20] have indicated the detrimental effects of excess body weight on bone; however, to our knowledge, only one of these studies [20] has taken into consideration menopausal status while examining this relationship. Therefore, the purpose of our study was to investigate the effects of obesity on bone considering the menopausal status of the rat.

The results of the current investigation indicate that mean BMD of L4 vertebrae was not altered in lean and obese intact Zucker rats whereas it was found to be significantly lower in lean and obese-Ovx Zucker rats. These observations are partially corroborated by the findings of Picherit at al. [21] who observed that 6-month lean-Ovx Zucker rats have lower femoral BMD than lean-sham Zucker rats; however, the effect of ovariectomy on femoral BMD of obese Zucker rats was not assessed. They also found the femoral BMD of obese Zucker rats to be distinctly lower than that of lean Zucker rats but similar to that of lean-Ovx Zucker rats, a discrepant result from our observations. Although unexpected, in the current study, obesity did not exacerbate or attenuate the effects of ovariectomy on BMD. Though the number of studies addressing the relationship between obesity and bone mass in Ovx-Zucker rats is limited, this relationship has been explored to a greater extent in C57BL/6J mice. For instance, Núñez et al. [20] reported that overweight-Ovx female C57BL/6J mice had higher whole body BMD when compared to lean-Ovx mice. In contrast, they found that very obese-Ovx mice (≥55% body fat) had markedly lower BMD than the overweight-Ovx mice. Their findings suggest that a threshold may exist in order for body weight to exert protective effects on bone. Hence, we postulate that there may be a U-shaped relationship between body weight and bone, though this needs further investigation. In addition, other investigators have reported that obesity induced by a high-fat diet significantly decreased femur [18] and lumbar BMD [19] in male C57BL/6J mice. In contrast, there are reports indicating that obesity induced by a high-fat diet increased spine BMD [22] but had no effect on whole body BMD in male mice [22, 23].

In terms of human studies, independent reports have indicated that high BMI was positively associated with greater whole-body and spine BMD in postmenopausal women [24, 25]. Case-control [5] and retrospective [2] studies have also demonstrated that obese postmenopausal women have greater femoral and lumbar spine BMD than nonobese postmenopausal women. The findings of a cross-sectional study by Castro et al. [3] reported that high BMI correlates with decreases and increases, by units, in the odds of low BMD in white and African-American women, respectively, suggesting a possible racial discrepancy. The relationship between BMI and BMD may be confounded by other factors. For instance, a study by Zhao et al. [12] reported that greater fat mass negatively correlates with BMD after correction for the mechanical loading effects of body weight in healthy adults. Altogether, much of the data presented from animal as well as human studies are inconclusive with respect to the effect of obesity on BMD.

With respect to bone trabecular properties, our results show that obesity did not affect any of the bone microstructural parameters in intact rats. Similar results were observed by Tamasi et al. [26]. Our findings also indicate that bone microstructural parameters were unfavorably affected by ovariectomy in lean and obese Zucker rats in comparison to those intact, with the exception of Tb.Th, which was preserved in lean-Ovx Zucker rats. Although there is a paucity of studies reporting the effect of obesity on bone microstructural parameters in Ovx Zucker rats, observations similar to ours have been noted in different strains of rats and mice. For instance, the preservation of Tb.Th observed in the lean-Ovx rats is in agreement with the findings of Yoshida et al. [27] who reported that bone loss in Ovx-Wistar rats is characterized by a loss of trabeculae rather than thinning of the trabeculae.

Interestingly, Tb.Th was found to be markedly lower in obese-Ovx Zucker rats when compared to the other groups suggesting that obesity has a negative effect on bone. In contrast to our findings, in male C57BL/6J mice, obesity induced by a high-fat diet did not affect Tb.Th but negatively altered all other microstructural parameters [19, 23]. These mouse studies examined the bone microarchitecture of femurs and lumbar vertebrae, respectively; yet in our study the tibiae were used. Thus, the difference in bone specimens may explain the discrepancy in our findings as it has been demonstrated that rates of trabecular bone loss are higher in the lumbar vertebrae than in the tibiae [28]. Nonetheless, a preservation of Tb.Th has been also observed in women [29, 30]. Particularly, the Os des Femmes de Lyon (OFELY) study [31] reported that obese postmenopausal women had significantly greater Tb.N, lower Tb.Sp, and similar Tb.Th at the distal tibia when compared to normal-weight women. Therefore, it is imperative that future studies investigate the effect of obesity on specific bones in order to make relevant comparisons.

The effects of ovariectomy on bone mass and microstructure in lean and obese Zucker rats were anticipated as the 6-month-old Ovx-rat is considered a rat model suitable to study postmenopausal bone loss [32]. Nonetheless, the validity of using obese-Ovx Zucker rats as a model of postmenopausal obesity remains unclear as these animals exhibit altered ovarian morphology, with decreased production of estrogen, and are not fertile [33]. Thus, one could argue that the obese intact Zucker rat is not the ideal model of premenopause in comparisons involving lean Zucker rats that exhibit 4-fold higher levels of estrogen than obese Zucker rats [33]. Indeed, estrogen is a necessary factor for bone growth and development as well as a regulator of bone turnover in mature bones [34]. The fact that bone mass did not differ between obese-Ovx and intact Zucker rats and their lean controls implicates adipose tissue as the source for the estrogen necessary to maintain bone mass in the absence of ovarian estrogen. It has been shown that most estrogen in postmenopausal women is produced in peripheral adipose tissue by aromatization of androstenedione to estrone [7, 35], especially in obese and overweight women. Studies by MacDonald et al. [35–38] demonstrated that the rate of conversion of androstenedione to estrone is increased by obesity. Another factor associated with obesity is low levels of sex hormone binding globulin which results in increased bioavailable estradiol [39].

Circulating leptin increases as body weight and fat mass increase [40, 41] which may be another contributing factor for the maintenance of bone mass in obese-Ovx Zucker rats observed in this study. Previous data suggest that serum leptin may play an important role in bone remodeling [11, 42]. Levels of leptin, which is a protein coded by the* ob/ob* gene and secreted by adipocytes, are increased in response to elevated estrogen levels [43, 44]. Obese Zucker rats have increased circulating leptin due to a leptin receptor mutation [45]. Increased aromatase activity contributes to increased estrogen synthesis in adipose tissue and may be an important factor in increasing leptin in obese Zucker rats. This may partially explain why bone mass was not significantly different between obese-Ovx and intact Zucker rats and their respective lean controls. In fact, the literature supports this notion as leptin deficient mice have been shown to have impaired bone growth [46]. In addition, administration of leptin to male mice has been shown to reduce bone fragility as denoted by increased work to failure and displacement in comparison to their controls [47].

The present study has several possible limitations. Cortical bone microstructural parameters as well as dynamic histomorphometry data and biomechanical properties were not evaluated. Future studies should assess these parameters as they may lead to a better understanding of the effects of obesity on bone in Ovx Zucker rats. Even though *Lepr* ^*fa*/*fa*^ rats are known to exhibit the obesity phenotype around 4-5 weeks of age and in the present study these rats were obese for approximately 22 weeks, we did not longitudinally examine weight and bone parameters which should be done in the future. Body composition (e.g., lean and fat mass) could provide additional insight into the relationship between obesity and bone in Ovx Zucker rats. Lastly, a larger sample size may have helped to detect significant differences among many of the parameters of interest. However, a sample size of six rats per group was used per other rat models as there were no similar studies available at the time of conducting the present study. Future studies should be conducted in this rat model using a larger sample size to enable the detection of significant differences for the parameters of interest.

In summary, the findings of animal and human studies regarding the effects of obesity on bone health are inconsistent and may be attributed to several factors. First, the majority of human studies report findings that are primarily derived from correlational analyses as opposed to controlled trials, thereby precluding a cause-and-effect relationship from being established. Second, existing animal studies vary in the use of species and strains as well as age, gender, and estrogenic environment. Third, various means of inducing obesity, for example, through genetic manipulation or high-fat diet, may influence the bone outcomes assessed. Therefore, the relationship between obesity and bone health cannot be established at this time. Collectively, aside from the adverse effects on Tb.Th, the findings of the present study do not show a directional relationship between obesity and bone health in Zucker rats.

## Figures and Tables

**Table 1:** Initial and final body weights of sham and ovariectomized lean and obese female Zucker rats.

	L/S	L/O	O/S	O/O
Body weight (g)				
Initial	107 ± 1.85^a^	101 ± 1.51^a^	167 ± 2.76^b^	160 ± 3.03^b^
Final	287 ± 5.02^a∗^	340 ± 3.55^b∗^	507 ± 9.55^c∗^	562 ± 8.78^d∗^

Values are expressed as mean ± SEM. Values that do not share the same superscript letters are significantly (*P* < 0.0001) different from each other. ∗*P* < 0.001 versus baseline. *n* = 6 rats/group. L/S, lean (*Lepr* ^*fa*/+^) sham-operated; L/O, lean (*Lepr* ^*fa*/+^) ovariectomized; O/S, obese (*Lepr* ^*fa*/*fa*^) sham-operated; and O/O, obese (*Lepr* ^*fa*/*fa*^) ovariectomized.

**Table 2:** Effects of ovariectomy and obesity on DXA bone parameters of fourth lumbar (L4) vertebrae.

	L/S	L/O	O/S	O/O	*P* value
BMA (cm^2^)	0.43 ± 0.012	0.41 ± 0.011	0.44 ± 0.017	0.43 ± 0.014	0.586
BMC (g)	0.097 ± 0.004^ab^	0.082 ± 0.003^a^	0.102 ± 0.005^b^	0.089 ± 0.005^ab^	0.030
BMD (g/cm^2^)	0.226 ± 0.004^a^	0.200 ± 0.002^b^	0.231 ± 0.004^a^	0.205 ± 0.007^b^	<0.001

Values are mean ± SEM; *n* = 10 rats/group. Values that do not share the same superscript letters are significantly (*P* < 0.05) different from each other. *n* = 6 rats/group. L/S, lean (*Lepr* ^*fa*/+^) sham-operated; L/O, lean (*Lepr* ^*fa*/+^) ovariectomized; O/S, obese (*Lepr* ^*fa*/*fa*^) sham-operated; and O/O, obese (*Lepr* ^*fa*/*fa*^) ovariectomized.
